# Gay affirmative practices among healthcare professionals in Poland and Spain: results of Health Exclusion Research in Europe (HERE) study

**DOI:** 10.3389/fpubh.2025.1568486

**Published:** 2025-04-22

**Authors:** Piotr Karniej, Anthony Dissen, Pablo del Pozo-Herce, Raúl Juárez-Vela, Antonio Martínez-Sabater, Vicente Gea-Caballero, Emmanuel Echaniz-Serrano, Iván Santolalla-Arnedo, Michał Czapla

**Affiliations:** ^1^Faculty of Finance and Management, WSB MERITO University in Wroclaw, Wrocław, Poland; ^2^Group of Research in Care and Health (GRUPAC), Faculty of Health Sciences, University of La Rioja, Logroño, Spain; ^3^School of Health Sciences, Stockton University, Galloway, NJ, United States; ^4^Research Group on Innovation in Health Care and Nursing Education (INcUidE), University of UNIE, Madrid, Spain; ^5^Nursing Care and Education Research Group (GRIECE), GIUV2019-456, Nursing Department, University of Valencia, Valencia, Spain; ^6^Care Research Group (INCLIVA), Hospital Clínico Universitario de Valencia, Valencia, Spain; ^7^Faculty of Health Sciences, Research Group Community Health and Care, International University of Valencia, Valencia, Spain; ^8^Sapienf (B53_23R) Research Group, Department of Physiatry and Nursing, Faculty of Health Sciences, University of Zaragoza, Zaragoza, Spain; ^9^Division of Scientific Research and Innovation in Emergency Medical Service, Department of Emergency Medical Service, Faculty of Nursing and Midwifery, Wroclaw Medical University, Wrocław, Poland; ^10^Institute of Heart Diseases, University Hospital, Wroclaw, Poland

**Keywords:** healthcare professionals, LGBTQ+ health, affirmative practice, cultural competency, attitude of health personnel

## Abstract

**Introduction:**

Healthcare professionals play a critical role in providing affirmative care to gay and lesbian patients. However, their attitudes and practices can vary significantly depending on cultural and educational contexts. This study aimed to evaluate differences in affirmative practices among healthcare professionals in Poland and Spain, focusing on their approach to these patient groups, utilizing the Gay Affirmative Practice (GAP) Scale, as well as identifying key factors influencing these practices.

**Methods:**

A cross-sectional study was conducted among healthcare professionals in Poland and Spain using the GAP Scale, which assesses beliefs and behaviors toward gay and lesbian patients. Data collection included 495 participants, with 205 from Spain and 290 from Poland. Descriptive statistics, Mann–Whitney tests, and multivariate regression analyses were used to identify factors associated with affirmative practices.

**Results:**

Spanish participants scored significantly higher on both the beliefs and behaviors scales compared to Polish participants (*p* < 0.001 and *p* = 0.009, respectively). Gender of healthcare providers was a significant factor in both groups, with women demonstrating more affirmative beliefs than men. In the Polish group, being male was associated with a decrease in the belief scale score by an average of 6.572 points (regression coefficient = −6.572, *p* < 0.001), while attending LGBT-related training 1–2 times was associated with an increase of 5.356 points on the belief scale (regression coefficient = 5.356, *p* = 0.039). No significant independent predictors were identified for behaviors in the Polish group, as all *p*-values exceeded 0.05.

**Conclusion:**

Spanish healthcare professionals showed more affirmative practices toward gay and lesbian patients than their Polish counterparts. Sex and gay and lesbian related training influenced beliefs, with male sex linked to lower affirmative practice in Poland. These findings highlight the need for systematic integration of gay and lesbian specific training into healthcare education programs to foster cultural competence and reduce disparities in patient care.

## 1 Introduction

The concept of “health inequalities” refers to the impact of factors such as wealth, education, occupation, race or ethnicity, sexual orientation, and other socio-cultural determinants on population health. These inequalities in healthcare contribute to poorer health outcomes in vulnerable social and cultural groups (1). Biases among health professionals can influence the quality of care; implicit prejudices related to race, age, gender identity, and sexual orientation generate inequalities in care and negatively affect health outcomes in vulnerable groups (2). Access to healthcare for gay and lesbian individuals can be hindered by various factors, including biases and discrimination from medical personnel. Research from different healthcare systems, particularly in the United States, has shown that race and ethnicity significantly contribute to healthcare disparities, often exacerbating barriers to affirming medical care (3). While this issue is widely studied in the U.S., there is a lack of research exploring how racial and ethnic factors influence LGBT healthcare experiences in Poland and Spain patients (4). A recent study by the European Union Agency for Fundamental Rights found that 16% of all LGBTI individuals, including transgender people, faced discrimination in healthcare settings, while among transgender respondents, this figure increased to 34% (5).

In recent years, the health of LGBT individuals has gained attention due to specific health concerns and significant mental health disparities compared to heterosexual and cisgender individuals (6–9). Gay and lesbian individuals face elevated rates of mental health issues, such as anxiety, depression, substance abuse, and suicidal risk. Additionally, they encounter significant barriers in accessing culturally competent healthcare, which may contribute to health disparities (10). They also experience higher rates of sexually transmitted infections (STIs), substance abuse, and mental health issues, contributing to a greater disease burden (11, 12).

Despite the need for inclusive healthcare, LGBT individuals encounter significant barriers to access, including discrimination, stigma, and a lack of specific competencies among healthcare professionals (8, 9, 13, 14). Meyer’s study found that LGBT individuals experience high levels of psychosocial stress due to societal discrimination and stigma, which negatively impacts mental health, increasing the incidence of anxiety, depression, and other disorders (15). Exposure to stigma is linked to adverse mental health outcomes, which constitute key sources of both morbidity and mortality in this population (10). Such inequalities in healthcare, often stemming from negative or discriminatory experiences with professionals and care providers, prompt many in the LGBT+ community to delay healthcare needs, which negatively impacts health. Research by Katz-Wise suggests that fear of discrimination makes LGBT+ individuals less likely to seek medical care (16, 17).

A major barrier to inclusive care is the lack of adequate training on sexual and gender diversity for healthcare professionals. Training institutions often omit specific LGBT-related content, perpetuating biases and exclusionary practices. This lack of training affects care quality and fosters an environment of stigma, impacting both professionals and patients, with negative repercussions on patient care experiences and trust in providers (18, 19). Healthcare professionals emphasize the importance of awareness, collaboration, and specific training on LGBT+ health issues (14). However, a general lack of knowledge and certain insecurities about treating this group remain, sometimes exacerbated by the pathologization of minority gender identities and sexual orientations. This suggests that proper training would allow healthcare professionals to approach LGBT+ patient care with greater safety and empathy. This would help address health disparities in this group and promote more inclusive and effective care.

The cultural and social context has been shown to play a key role in shaping health professionals’ attitudes and practices when they are working with the patients that they serve. In countries such as Poland and Spain, regulatory frameworks, social climate, and public perception of LGBT+ individuals vary, influencing affirmative practices among professionals. While Spain has made significant progress in implementing laws protecting LGBT+ rights, Poland faces challenges in accepting and normalizing these rights, potentially affecting healthcare professionals’ readiness to adopt affirmative practices (20, 21).

The aim of this study was to evaluate the differences in affirmative practices toward gay and lesbian patients among healthcare professionals in Poland and Spain using the Gay Affirmative Practice Scale, and to identify which key factors were shown to have the greatest force of influencing these practices.

## 2 Methodology

### 2.1 Study design and participants

This cross-sectional study was conducted between February 2023 and August 2024 among healthcare professionals in Poland and Spain. Participants included a range of healthcare professionals and students in fields such as nursing, medicine, and the allied health professions. Eligibility criteria required participants to be at least 18 years old, proficient in Polish or Spanish, and active in a medical profession or education program.

### 2.2 Data collection and tools

Data were collected via an online platform Webankieta (22), using an anonymous, selfadministered questionnaire, which was distributed through targeted campaigns on social media platforms, including Facebook and Instagram. Participants were informed about the study’s purpose, objectives, and procedures, and provided informed consent before participating. The survey was designed to ensure the integrity of responses by utilizing IP filtering to prevent multiple submissions from the same user. It is worth emphasizing that this process was fully automated, and researchers had no direct access to individual IP addresses.

The questionnaire comprised two sections. The first section gathered demographic information, including age, gender, place of residence, marital status, profession, and participation in LGBT-related training within the last 5 years. In the interest of analytical clarity, only heterosexual respondents were included in further analyses. Additionally, certain demographic responses were consolidated: individuals who were divorced or widowed were grouped together, and only legally recognized marriages were considered.

The second section utilized the Gay Affirmative Practice (GAP) Scale, a psychometric tool originally developed by Catherine Crisp to evaluate healthcare providers’ beliefs and behaviors in their work with lesbian and gaypatients (23). For this study, the Polish (GAP-PL) (24) and Spanish (GAP-ES) (25) versions of the GAP Scale, previously adapted by Karniej et al., were used, both developed as part of the Health Exclusion Research in Europe (HERE) project (26).

The Polish version (GAP-PL) demonstrated high internal consistency, with Cronbach’s alpha values ranging from 0.936 to 0.949 across subscale domains, and a McDonald’s omega coefficient of 0.963, indicating excellent reliability (24). The Spanish version (GAP-ES) also showed strong internal consistency, with Cronbach’s alpha values of 0.915 for the beliefs subscale and 0.902 for the behaviors subscale, and a McDonald’s omega coefficient of 0.942 (25). Each item on the GAP Scale is scored from 15 to 75, with higher scores reflecting more affirmative practices. The robust reliability of these versions supports their use in cross-cultural comparisons between Polish and Spanish healthcare professionals.

### 2.3 Statistical analysis

The mean, standard deviation, median, quartiles, and range were calculated for quantitative variables. For categorical variables, absolute and relative frequencies (*N* and %) were presented. To compare categorical variables between groups, the chi-squared test was applied (with Yates correction for 2×2 tables), or Fisher’s exact test was used when expected values were low. For quantitative variable comparisons between two groups, the Mann–Whitney U test was employed, while comparisons across three or more groups were performed using the Kruskal-Wallis test, followed by Dunn’s post-hoc test if needed. Spearman’s correlation coefficient was used to examine relationships between two quantitative variables. Multiple linear regression was utilized to analyze the influence of various predictors on quantitative outcomes, with regression parameters and 95% confidence intervals reported. A significance level of 0.05 was set for all analyses. Statistical analyses were carried out using R software, version 4.4.1 (27).

## 3 Results

### 3.1 Characteristics of the study population

The study population comprised healthcare professionals from Spain and Poland, with notable demographic differences between the two groups. The proportion of male respondents was higher in the Polish group, whereas the Spanish group was older on average, with a significantly higher mean age (*p* < 0.001). Polish participants were more likely to reside in larger cities (*p* < 0.001). In terms of marital status, Polish respondents were more frequently single, while Spanish participants reported higher rates of formal partnerships and marriages (*p* < 0.001). Professionally, a higher percentage of nurses/midwives were found in the Spanish group, whereas the Polish group had a greater proportion of physicians, students, and other healthcare professionals (*p* < 0.001). Additionally, participation in LGBT-related trainings was more frequent among Spanish respondents (*p* < 0.001). Full data regarding the characteristics of the study group are presented in Table 1.

**Table 1:** Characteristics of the study population.

Parameter		Spain (*N* = 205)	Poland (*N* = 290)	Total (*N* = 495)	*p*
Gender	Female	132 (64.39%)	156 (53.79%)	288 (58.18%)	*p* = 0.024 *
	Male	73 (35.61%)	134 (46.21%)	207 (41.82%)	
Age [years]	Mean (SD)	38.96 (9.58)	31.4 (8.89)	34.53 (9.9)	*p* < 0.001 *
	Median (quartiles)	39 (32–47)	30 (25–36)	33 (27–41)	
	Range	18–63	18–63	18–63	
	n	205	290	495	
Place of residence	Village	28 (13.66%)	36(12.41%)	64 (12.93%)	*p* = 0.002 *
	City of up to 20,000 inhabitants	5(2.44%)	17(5.865%)	22(4.44%)	
	City of between 20,000 and 100,000 inhabitants	37	41 (14.14%)	78 (15.76%)	
	City of between 100,000 and 500,000 inhabitants	51 (24.88%)	40 (13.79%)	91 (18.38%)	
	City of more than 500,000	84 (40.98%)	156 (53.79%)	240 (48.48%)	
Sexual orientation	Heterosexual	137 (66.83%)	185 (63.79%)	322 (65.05%)	*p* = 0.547
	Homosexual	68 (33.17%)	105 (36.21%)	173 (34.95%)	
Marital status	Single	37(18.05%)	87 (30.00%)	124 (25.05%)	*p* < 0.001 *
	In marriage	83(40.49%)	61 (21.03%)	144 (29.09%)	
	In a (formal) partnership	71 (34.63%)	5 (1.72%)	76 (15.35%)	
	In a non-formalised relationship	2 (0.98%)	130 (44.83%)	132 (26.67%)	
	Divorce/Separation	10 (4.88%)	7 (2.41%)	17 (3.43%)	
	Widower / Widow	2 (0.98%)	0 (0.00%)	2 (0.40%)	
Profession	Nurse/midwife	149 (72.68%)	67 (23.10%)	216(43.64%)	*p* < 0.001 *
	Physician	21(10.24%)	82 (28.28%)	103(20.81%)	
	Other health profession	27 (13.17%)	85 (29.31%)	112 (22.63%)	
	Student	8 (3.90%)	56 (19.31%)	64 (12.93%)	
LGBT-related trainings (conferences, webinars) in last 5 years	Never	126 (61.46%)	235 (81.03%)	361 (72.93%)	*p* < 0.001 *
	1–2 times	60 (29.27%)	43 (14.83%)	103 (20.81%)	
	3–5 times	13 (6.34%)	7 (2.41%)	20 (4.04%)	
	More than 5 times	6 (2.93%)	5 (1.72%)	11 (2.22%)	

*p—Qualitative variables: chi-squared or Fisher’s exact test. Quantitative variables: Mann–Whitney test.*

* Statistically significant (*p* < 0.05).

### 3.2 Results of GAP comparison between Poland and Spain

The GAP questionnaire evaluated respondents’ beliefs and behaviors towards gay and lesbian patients, with scores ranging from 15 to 75 on each scale. Higher scores indicate a more affirmative approach, and analysis was limited solely to heterosexual participants. The Spanish group scored significantly higher on the beliefs scale compared to the Polish group (*p* < 0.001), indicating more affirmative attitudes towards gay and lesbian patients. Similarly, the Spanish group scored higher on the behaviors scale than the Polish group (*p* = 0.009), reflecting more affirmative behavioral responses. Full data regarding the GAP scale scores are presented in Table 2.

**Table 2:** Results of GAP comparison between Poland and Spain.

GAP	Country	*N*	Mean	SD	Median	Min	Max	Q1	Q3	*p*
Beliefs	Spain	137	67.93	7.32	70	47	75	64	75	*p* < 0.001 *
	Poland	185	64.54	9.3	66	15	75	60	72	
Behaviors	Spain	137	55.38	12.03	57	15	75	47	65	*p* = 0.009 *
	Poland	185	50.66	14.93	73	15	75	44	61	

*p—Mann–Whitney test; SD, standard deviation; Q1, lower quartile; Q3, upper quartile.*

* Statistically significant (*p* < 0.05).

### 3.3 GAP scale scores in the Spanish group

In the Spanish heterosexual group, gender differences were observed on the belief scale, with women scoring significantly higher than men (*p* = 0.037). However, no statistically significant differences were found across other demographic variables, including place of residence, marital status, profession, and frequency of LGBT-related training in the past 5 years (all *p*-values > 0.05).

For marital status, widowed and divorced individuals were analyzed as a combined category, and distinctions between marriage to a man or woman were not considered, as the analysis was conducted separately for heterosexual and homosexual individuals. Additionally, participants who attended LGBT-related trainings more than five times were grouped with those who attended three to five times.

Regarding age, no significant correlations were identified with either the beliefs or behaviors domains. Specifically, Spearman’s correlation coefficient for age and beliefs was r = −0.019, *p* = 0.826, and for age and behaviors, r = 0.01, *p* = 0.908. Full data for these analyses are presented in Table 3.

**Table 3:** Analysis of GAP scale scores by characteristics in the Spanish group.

GAP*	Gender	*N*	Mean	SD	Median	Min	Max	Q1	Q3	*p*
Beliefs	Female	122	68.34	7.2	71	47	75	65	75	0.037
	Male	15	64.53	7.69	66	49	75	61	69	
Behaviors	Female	122	55.77	12.34	57	15	75	48	65	0.137
	Male	15	52.2	8.73	49	39	66	44.5	60	

GAP**	Place of residence	*N*	Mean	SD	Median	Min	Max	Q1	Q3	*p*
Beliefs	Village	24	68.12	68.12	71.5	47	75	64	74.25	0.225
	City of up to 20,000 inhabitants	3	63.67	63.67	65	58	68	61.5	66.5	
	City of between 20,000 and 100,000 inhabitants	27	69.59	69.59	72	56	75	61.5	75	
	City of between 100,000 and 500,000 inhabitants	34	68.68	68.68	71	51	75	66	75	
	City of more than 500,000 inhabitants	49	66.65	66.65	68	49	75	63	73	
Behaviors	Village	24	55.83	55.83	56	36	75	48.5	66	0.99
	City of up to 20,000 inhabitants	3	56.67	56.67	58	49	63	53.5	60.5	
	City of between 20,000 and 100,000 inhabitants	27	55.41	55.41	55	15	75	50	65	
	City of between 100,000 and 500,000 inhabitants	34	54.62	54.62	58.5	20	73	44.25	62.75	
	City of more than 500,000 inhabitants	49	55.59	55.59	59	15	73	47	65	

GAP**	Marital status	*N*	Mean	SD	Median	Min	Max	Q1	Q3	*p*
Beliefs	Single	19	66.42	7.31	68	49	75	65	71.5	0.743
	In marriage	69	68.22	7.2	70	47	75	65	74	
	In a (formal) partnership	39	68.08	7.52	71	52	75	62.5	75	
	In a non-formalised relationship	2	69	8.49	69	63	75	66	72	
	Divorced/Separated/Widowed	8	68	8.73	69.5	49	75	66.25	75	
Behaviors	Single	19	54.74	11.76	58	34	72	47	65	0.718
	In marriage	69	54.33	13.5	54	15	75	46	64	
	In a (formal) partnership	39	56.26	10.05	57	36	75	48.5	64.5	
	In a non-formalised relationship	2	59.5	0.71	59.5	59	60	59.25	59.75	
	Divorced/Separated/Widowed	8	60.62	9.12	63.5	43	71	57.25	66.25	

GAP**	Profession	*N*	Mean	SD	Median	Min	Max	Q1	Q3	*p*
Beliefs	Nurse/midwife	110	68.21	7.15	70.5	47	75	65	74	0.434
	Physician	4	58.5	11.62	55	49	75	51.25	62.25	
	Other health profession	18	68.11	6.88	69	55	75	65	74.75	
	Student	5	68.6	5.94	66	63	75	64	75	
Behaviors	Nurse/midwife	110	55.1	11.79	56	15	75	47	64	0.413
	Physician	4	50.25	7.09	49	43	60	47.5	51.75	
	Other health profession	18	57.33	15.17	62.5	15	72	48.75	68.25	
	Student	5	58.6	7.23	59	49	69	56	60	

GAP**	LGBT-related trainings in last 5 years	*N*	Mean	SD	Median	Min	Max	Q1	Q3	*p*
Beliefs	Never	85	67.15	7.54	68	47	75	63	74	0.192
	1–2 times	44	69.02	6.96	71.5	52	75	65.75	75	
	More than 2 times	8	70.12	6.42	72	56	75	68	75	
Behaviors	Never	85	53.98	13.09	55	15	75	45	64	0.304
	1–2 times	44	57.43	9.91	59	36	73	49	65	
	More than 2 times	8	59	9.24	60.5	47	69	49.75	67.5	

*p—Mann–Whitney test; **p—Kruskal-Wallis test; SD, standard deviation; Q1, lower quartile; Q3, upper quartile.

### 3.4 GAP scale scores in the polish group

In the Polish heterosexual group, belief scale scores were significantly higher among women than men (*p* = 0.009). No statistically significant relationships were found across other demographic variables, including age, place of residence, marital status, profession, or frequency of LGBT-related training in the past 5 years (all *p*-values > 0.05). For age, Spearman’s correlation coefficient was r = −0.018, *p* = 0.809 for beliefs and r = −0.02, *p* = 0.788 for behaviors. Full data for these analyses are presented in Table 4.

**Table 4:** Analysis of GAP scale scores by characteristics in the Polish group.

GAP*	Gender	*N*	Mean	SD	Median	Min	Max	Q1	Q3	*p*
Beliefs	Female	149	65.58	8.27	66	16	75	61	63	0.009
	Male	36	60.19	11.9	62.5	15	75	53.75	68	
Behaviors	Female	149	51.46	14.81	54	15	75	45	61	0.098
	Male	36	47.33	15.17	49	15	73	39	59	

GAP**	Place of residence	*N*	Mean	SD	Median	Min	Max	Q1	Q3	*p*
Beliefs	Village	32	63.91	11.62	65.5	15	75	59.5	74	0.89
	City of up to 20,000 inhabitants	14	64.5	9.22	67	40	75	59.5	70.5	
	City of between 20,000 and 100,000 inhabitants	34	64.03	7.52	62	49	75	58.25	69.75	
	City of between 100,000 and 500,000 inhabitants	18	65.94	7.83	67	47	75	61	72.75	
	City of more than 500,000 inhabitants	87	64.68	9.45	66	16	75	60.5	71.5	
Behaviors	Village	32	51.78	15.7	54.5	15	72	48	61.25	0.729
	City of up to 20,000 inhabitants	14	51.71	15.57	54.5	18	75	43.75	61.25	
	City of between 20,000 and 100,000 inhabitants	34	48.38	14.23	47	15	73	41	59	
	City of between 100,000 and 500,000 inhabitants	18	49.17	19.43	49.5	15	75	40.25	64	
	City of more than 500,000 inhabitants	87	51.28	13.99	53	15	73	44.5	61	

GAP**	Marital status	*N*	Mean	SD	Median	Min	Max	Q1	Q3	*p*
Beliefs	Single	62	65.15	9.91	67	15	75	61	72.75	0.488
	In marriage	59	64.68	8.26	65	40	75	60	71	
	In a (formal) partnership	1	75	–	75	75	75	75	75	
	In a non-formalised relationship	56	63.43	9.97	64.5	16	75	59	70	
	Divorced/Separated/Widowed	7	65.29	7.13	63	54	75	62	70.5	
Behaviors	Single	62	51.71	14.57	53.5	15	75	43	61.75	0.965
	In marriage	59	50.32	14.81	53	15	74	44	59	
	In a (formal) partnership	1	48	---	48	48	48	48	48	
	In a non-formalised relationship	56	49.48	16.15	54	15	75	43.25	61	
	Divorced/Separated/Widowed	7	54	11.2	52	36	68	49	62	

GAP**	Profession	*N*	Mean	SD	Median	Min	Max	Q1	Q3	*p*
Beliefs	Nurse/midwife	40	63.98	7.9	65.5	40	75	58.75	70	0.857
	Physician	36	64.33	11.45	65.5	16	75	60.75	73	
	Other health profession	65	65.11	7.35	64	52	75	60	72	
	Student	44	64.36	11.22	67	15	75	59.75	73	
Behaviors	Nurse/midwife	40	51.7	15.24	52	15	75	44.75	63	0.609
	Physician	36	52.53	12.79	55.5	15	72	43	61	
	Other health profession	65	48.45	15.55	51	15	75	44	59	
	Student	44	51.45	15.44	54	17	73	43.75	62.5	

GAP**	LGBT-related trainings in last 5 years	*N*	Mean	SD	Median	Min	Max	Q1	Q3	*p*
Beliefs	Never	168	64.17	9.45	65	15	75	59	71.25	0.149
	1–2 times	15	68	7.07	68	48	75	66	73.5	
	More than 2 times	2	69	8.49	69	63	75	66	72	
Behaviors	Never	168	50.4		53	15	75	43	61	0.548
	1–2 times	15	52.07	14.1	53	15	73	45.5	61	
	More than 2 times	2	61.5	12.02	61.5	53	70	57.25	65.75	

*p—Mann–Whitney test; **p—Kruskal-Wallis test; SD, standard deviation; Q1, lower quartile; Q3, upper quartile.

### 3.5 Multivariate analysis

In the Spanish group, the multivariate linear regression model indicated that none of the analyzed characteristics were significant independent predictors of scores on either the beliefs or behaviors scales, as all *p*-values were greater than 0.05. Table 5. Multivariate Analysis of Independent Predictors for GAP Scale Scores in the Spanish Group.

**Table 5:** Multivariate analysis of independent predictors for GAP scale scores in the Spanish group.

Trait	Level	Beliefs	Behaviors
Gender	Female	ref.	ref.
	Male	−2.799 (−7.055 to 1.457), *p* = 0.2	−3.617 (−10.799 to 3.564), *p* = 0.326
Age [years]		−0.028 (−0.196 to 0.14), *p* = 0.746	−0.025 (−0.308 to 0.258), *p* = 0.862
Place of residence	Village	ref.	ref.
	City of up to 20,000 inhabitants	−1.768 (−11.457 to 7.922), *p* = 0.721	2.3 (−14.049 to 18.649), *p* = 0.783
	City of between 20,000 and 100,000 inhabitants	1.379 (−2.743 to 5.501), *p* = 0.513	−0.448 (−7.403 to 6.507), *p* = 0.9
	City of between 100,000 and 500,000 inhabitants	0.518 (−3.435 to 4.472), *p* = 0.798	−1.615 (−8.286 to 5.055), *p* = 0.636
	City of more than 500,000 inhabitants	−0.967 (−4.661 to 2.727), *p* = 0.609	−0.223 (−6.455 to 6.01), *p* = 0.944
Marital status	Single	ref.	ref.
	In marriage	2.468 (−1.773 to 6.708), *p* = 0.256	1.174 (−5.98 to 8.329), *p* = 0.748
	In a (formal) partnership	1.314 (−2.893 to 5.522), *p* = 0.542	1.49 (−5.609 to 8.589), *p* = 0.682
	In a non-formalised relationship	2.472 (−8.675 to 13.62), *p* = 0.665	5.145 (−13.664 to 23.954), *p* = 0.593
	Divorced/Separated/Widowed	3.73 (−2.86 to 10.319), *p* = 0.269	9.027 (−2.091 to 20.145), *p* = 0.114
Profession	Nurse/midwife	ref.	ref.
	Physician	−8.267 (−16.62 to 0.085), *p* = 0.055	−5.733 (−19.826 to 8.36), *p* = 0.427
	Other health profession	0.259 (−3.663 to 4.181), *p* = 0.897	1.938 (−4.679 to 8.555), *p* = 0.567
	Student	1.533 (−5.83 to 8.897), *p* = 0.684	3.086 (−9.338 to 15.51), *p* = 0.627
LGBT-related trainings (conferences, webinars) in last 5 years	Never	ref.	ref.
	1–2 times	2.491 (−0.307 to 5.288), *p* = 0.084	3.613 (−1.107 to 8.334), *p* = 0.136
	More than 2 times	2.701 (−2.84 to 8.242), *p* = 0.341	5.469 (−3.879 to 14.818), *p* = 0.254

In the Polish heterosexual group, the multivariate linear regression model showed that being male decreases the belief scale score by an average of 6.572 points, as the regression coefficient is −6.572 (*p* < 0.001). Additionally, attending LGBT-related training 1–2 times increases the belief scale score by an average of 5.356 points, as the regression coefficient is 5.356 (*p* = 0.039). No significant independent predictors were identified for the behavior scale (all *p*-values > 0.05). Full data for these analyses are presented in Table 6.

**Table 6:** Multivariate analysis of independent predictors for GAP scale scores in the Polish group.

Trait	Level	Beliefs	Behaviors
Gender	Female	ref.	ref.
	Male	−6.572 (−10.109 to 3.035), *p*	−4.315 (−10.168 to 1.538), *p* = 0.15
Age [years]		−0.085 (−0.276 to 0.105), *p* = 0.381	−0.126 (−0.441 to 0.189), *p* = 0.434
Place of residence	Village	ref.	ref.
	City of up to 20,000 inhabitants	1.705 (−4.346 to 7.756), *p* = 0.582	1.321 (−8.693 to 11.335), *p* = 0.796
	City of between 20,000 and 100,000 inhabitants	0.721 (−3.774 to 5.216), *p* = 0.754	−3.011 (−10.449 to 4.428), *p* = 0.429
	City of between 100,000 and 500,000 inhabitants	1.588 (−3.888 to 7.065), *p* = 0.571	−2.538 (−11.601 to 6.525), *p* = 0.584
	City of more than 500,000 inhabitants	0.59 (−3.615 to 4.794), *p* = 0.784	−0.327 (−7.285 to 6.63), *p* = 0.927
Marital status	Single	ref.	ref.
	In marriage	0.413 (−3.476 to 4.303), *p* = 0.835	0.002 (−6.434 to 6.438), *p* = 0.999
	In a (formal) partnership	8.598 (−9.755 to 26.951), *p* = 0.36	−2.231 (−32.602 to 28.14), *p* = 0.886
	In a non-formalised relationship	−1.835 (−5.274 to 1.604), *p* = 0.297	−2.341 (−8.031 to 3.35), *p* = 0.421
	Divorced/Separated/Widowed	−0.302 (−8.081 to 7.477), *p* = 0.939	1.767 (−11.106 to 14.64), *p* = 0.788
Profession	Nurse/midwife	ref.	ref.
	Physician	1.661 (−2.946 to 6.268), *p* = 0.481	1.637 (−5.987 to 9.261), *p* = 0.674
	Other health profession	1.589 (−2.286 to 5.465), *p* = 0.423	−3.333 (−9.746 to 3.08), *p* = 0.31
	Student	−0.034 (−4.657 to 4.588), *p* = 0.988	−1.188 (−8.837 to 6.461), *p* = 0.761
LGBT-related trainings (conferences, webinars) in last 5 years	Never	ref.	ref.
	1–2 times	5.356 (0.306 to 10.407), *p* = 0.039 *	2.771 (−5.587 to 11.129), *p* = 0.517
	More than 2 times	2.611 (−10.402 to 15.624), *p* = 0.69	9.161 (−12.373 to 30.695), *p* = 0.406

## 4 Discussion

This study evaluated the differences in affirmative practices toward gay and lesbian patients among healthcare professionals in Poland and Spain using the GAP, highlighting the factors that most influence these practices, and their implications for addressing health inequalities in these two healthcare systems (28, 29). The findings reveal significant differences between the two countries, consistent with prior research showing how cultural and legislative environments shape healthcare professionals’ willingness to adopt inclusive practices (21). Demographic analysis indicated notable disparities. Spanish professionals were older on average, with a higher proportion of women, suggesting a potential link between professional experience and sensitivity to sexual diversity (8, 9). In contrast, the predominance of male and single professionals in Poland may reflect sociocultural factors that limit openness toward inclusive practices (30–32). Similar issues have been observed globally, where discrimination in LGBT care is linked to inadequate training among professionals and educators (33).

The results of the residency analysis also indicated that, while in Spain health professionals are more evenly distributed in urban and rural areas, in Poland there is a greater concentration in large cities. This difference may have important implications for the accessibility of health services in rural settings in both countries (34–36), as studies have shown that rural areas often present greater barriers to inclusive healthcare. This issue disproportionately affects lesbian and gay individuals (30, 31), who may experience stigma in their environment, leading to a lower willingness to seek healthcare services in their communities.

Along the same lines, the study revealed significant differences in LGBT-related training among professionals in both countries. A total of 61.46% of Spanish participants and 81.03% of Polish participants reported never having participated in such training. Among those who did, most attended events only once or twice in the past 5 years. These findings emphasize the persistent link between insufficient sexual diversity training and the stigmas affecting the healthcare of gay and lesbian individuals. According to the European Union Agency for Fundamental Rights, 34% of transgender individuals report discrimination in healthcare settings, while 46% of LGBT individuals avoid disclosing their identity to providers out of fear of discrimination (37). A lack of training on sexual and gender diversity issues remains a recurring barrier to developing competencies necessary for inclusive care (24–26, 28). Furthermore, insufficient training undermines the perceived quality of care and erodes trust between LGBT patients and healthcare providers, contributing to gaps in inclusive health services (38).

The frequency of training emerged as a positive predictor of affirmative attitudes in Poland, demonstrating that LGBT-specific competency training fosters inclusivity and reduces the risk of pathologizing sexual orientation, particularly in environments with lower social acceptance of sexual minorities (30, 32, 39). Limited access to such training appears to hinder healthcare professionals’ ability to provide respectful and affirming care (15, 39–42), especially in Polish settings where cultural sensitivity toward sexual diversity remains a significant challenge (32, 43). Comprehensive training programs could mitigate implicit biases and improve health outcomes, particularly for vulnerable groups who often encounter greater barriers to accessing affirmative healthcare (17, 44).

The GAP scale assessment revealed that Spanish healthcare professionals scored significantly higher than their Polish counterparts on both affirmative beliefs (mean: 67.93 vs. 64.54) and behaviors (mean: 55.38 vs. 50.66), with results showing statistical significance (*p* < 0.001). This disparity likely reflects the cultural and legislative differences between the two countries.

Spain’s legal and social framework supports LGBT rights, fostering a more inclusive and affirmative clinical environment. In contrast, Poland’s conservative policies and attitudes may hinder the ability of healthcare professionals to provide empathetic and inclusive care, posing a significant barrier to equality in the healthcare setting. For example, Poland does not legally recognize same-sex unions and has implemented restrictions on discussions of LGBT issues in schools and public institutions (4, 15, 30, 32, 39). The significant difference in the percentage of nonformalized relationships between Spain and Poland in our study (0.98% vs. 44.83%) may be explained by the fact that Spain has legalized same-sex marriage, while Poland still does not recognize same-sex unions, though legislative efforts have been initiated in recent years (4).

A detailed analysis of subgroups within the Spanish population revealed that while women scored higher on the belief scale compared to men, no significant differences were observed on the behavior scale or other demographic variables, such as place of residence or marital status. This suggests that, despite some gender-based variations, affirmative practices in Spain are generally consistent across demographics, likely reflecting the country’s supportive social and legal context for LGBT rights, which fosters a broadly affirmative attitude among healthcare professionals. In Poland, gender differences were more pronounced, with women showing significantly higher affirmative beliefs compared to men. This disparity may be shaped by sociocultural factors, including traditional gender roles and conservative norms, which could limit men’s openness to inclusivity and diversity. In many conservative societies, masculinity is often associated with rigid gender expectations and heteronormativity, which may lead to lower acceptance of sexual minorities and reluctance to engage in affirmative practices (45, 46). Notably, LGBT-related training in Poland was positively associated with affirming beliefs, underscoring the potential of such training to address these barriers and enhance cultural competence among healthcare professional (32, 47).

It is noteworthy that a study conducted in China identified nursing educators as the group with the lowest scores in attitudes and knowledge regarding LGBT issues, compared to nursing students and practicing nurses (48). This finding is particularly concerning, as these educators are responsible for training future healthcare professionals. This highlights the urgent need for targeted training initiatives aimed at educators to enhance their cultural competence. In the United States, Italy and Spain, the Attitudes Toward LGBT People Scales have been used to assess attitudes toward LGBT people among university students, finding that social contact can reduce prejudice. In Brazil, a cross-sectional online survey during the COVID-19 pandemic characterized the LGBT population and found high levels of violence and discrimination. In Europe, the European Social Survey has been used to compare health and wellbeing between individuals in same-sex and opposite-sex partnerships, showing significant disparities (49, 50). In summary, studies indicate that social contact may reduce prejudice. These studies indicate that discrimination and lack of competence in health care are common problems for LGBT people.

Such findings underscore the global nature of this issue, affecting diverse cultures and regions. Implementing transformative measures, such as diversity and inclusion training, has proven effective; interventions with nursing students have demonstrated significant improvements in GAP scores post-training. Similarly, self-reflection exercises have been shown to enhance affirmative attitudes (51).

## 5 Limitations

This study has several limitations. The cross-sectional design prevents the establishment of causal relationships between variables. The reliance on surveys for data collection may have excluded certain healthcare professionals, particularly those in rural areas with limited internet access, potentially skewing the sample. Additionally, a larger and more diverse cohort could have offered a more comprehensive understanding of affirmative attitudes across different subgroups. The study’s quantitative methodology also restricted the ability to investigate the underlying motivations or barriers influencing affirmative practices. Incorporating qualitative methods in future research could provide richer insights and enhance the interpretation of these findings.

## 6 Conclusion

Healthcare professionals in Spain demonstrated significantly more affirmative practices toward gay and lesbian patients than their Polish counterparts, as indicated by higher scores on the Gay Affirmative Practice Scale. Sex emerged as an influential factor, with female professionals exhibiting more affirmative beliefs in both countries. In Poland, male sex correlated with lower scores on the beliefs scale, while participation in gay and lesbian-related training was associated with improved affirmative practices. Notably, no significant predictors were identified for behaviors in the Polish group.

Given the significant impact of gay and lesbian-related training on healthcare professionals’ affirmative beliefs, we strongly recommend the integration of structured and mandatory gay and lesbian-focused education modules into medical and allied health curricula. Such training should include practical and experiential learning components, such as simulations, role-playing, and patient interactions, to ensure effective knowledge transfer and promote inclusivity in clinical practice. By addressing gaps in cultural competence, these interventions can help reduce healthcare disparities and foster equitable care for gay and lesbian patients.

## Data Availability

The raw data supporting the conclusions of this article will be made available by the authors, without undue reservation.

## References

[1] Nowaskie DZ Najam S. Lesbian, gay, bisexual, and/or transgender (LGBT) cultural competency across the intersectionalities of gender identity, sexual orientation, and race among healthcare professionals. PLoS One. (2022) 17:e0277682. doi: 10.1371/journal.pone.0277682, PMID: 36367906 PMC9651569

[2] Urrutia MT Cianelli R. Disparidad en Salud: Un Fenómeno Multidimensional. Available online at: https://pmc.ncbi.nlm.nih.gov/articles/PMC3349157/ (Accessed November 17, 2024). 10.1891/1540-4153.8.1.23 PMC3349157 22581053

[3] Macias-Konstantopoulos WL Collins KA Diaz R Duber HC Edwards CD Hsu AP. Race, healthcare, and health disparities: a critical review and recommendations for advancing health equity. West J Emerg Med. (2023) 24:906–18. doi: 10.5811/westjem.58408, PMID: 37788031 PMC10527840

[4] Jesus S. Annual review 2025 | ILGA-Europe. (2025). Available online at: https://www.ilga-europe.org/report/annual-review-2025/ (Accessed March 17, 2025)

[5] Council of Europe. Report highlights inadequate healthcare access for LGBTI people, recommends solutions. Portal. Available online at: https://www.coe.int/en/web/committee-antidiscrimination-diversity-inclusion/-/report-highlights-inadequate-healthcare-access-for-lgbti-people-recommends-solutions (Accessed November 17, 2024)

[6] Lucassen MF Stasiak K Samra R Frampton CM Merry SN. Sexual minority youth and depressive symptoms or depressive disorder: a systematic review and meta-analysis of population-based studies. Aust N Z J Psychiatry. (2017) 51:774–87. doi: 10.1177/0004867417713664, PMID: 28565925

[7] Pellicane MJ Ciesla JA. Associations between minority stress, depression, and suicidal ideation and attempts in transgender and gender diverse (TGD) individuals: systematic review and meta-analysis. Clin Psychol Rev. (2022) 91:102113. doi: 10.1016/j.cpr.2021.102113, PMID: 34973649

[8] Ho SH Shamsudin AH Liow JW Juhari JA Ling SA Tan K. Mental healthcare needs and experiences of LGBT+ individuals in Malaysia: utility, enablers, and barriers. Healthcare. (2024) 12:998. doi: 10.3390/healthcare12100998, PMID: 38786409 PMC11120647

[9] Rees SN Crowe M Harris S. The lesbian, gay, bisexual and transgender communities’ mental health care needs and experiences of mental health services: an integrative review of qualitative studies. J Psychiatr Ment Health Nurs. (2021) 28:578–89. doi: 10.1111/jpm.12720, PMID: 33295065

[10] Adelson SL Walker-Cornetta E Kalish N. LGBT youth, mental health, and spiritual care: Psychiatric collaboration with health care chaplains. J Am Acad Child Adolesc Psychiatry. (2019) 58:651–655. doi: 10.1016/j.jaac.2019.02.009 31229180

[11] Han BH Duncan DT Arcila-Mesa M Palamar JJ. Co-occurring mental illness, drug use, and medical multimorbidity among lesbian, gay, and bisexual middle-aged and older adults in the United States: a nationally representative study. BMC Public Health. (2020) 20:1123. doi: 10.1186/s12889-020-09210-6, PMID: 32746891 PMC7401198

[12] Everett BG. Sexual orientation disparities in sexually transmitted infections: examining the intersection between sexual identity and sexual behavior. Arch Sex Behav. (2013) 42:225–36. doi: 10.1007/s10508-012-9902-1, PMID: 22350122 PMC3575167

[13] Cronin TJ Pepping CA Lyons A. Mental health service use and barriers to accessing services in a Cohort of transgender, gender diverse, and non-binary adults in Australia. Sex Res Soc Policy. (2023) 1–14. doi: 10.1007/s13178-023-00866-4

[14] Talbot J Finlay F. Empowering healthcare professionals with health promotion information for transgender adolescents. Arch Dis Child Educ Pract Ed. (2023) 108:158–62. doi: 10.1136/archdischild-2022-324744 36347600

[15] Meyer IH. Prejudice, social stress, and mental health in lesbian, gay, and bisexual populations: conceptual issues and research evidence. Psychol Bull. (2003) 129:674–97. doi: 10.1037/0033-2909.129.5.674, PMID: 12956539 PMC2072932

[16] Dale VH Philomin R. Educating for quality transgender health care: a survey…: Education for health. Available online at: https://journals.lww.com/edhe/fulltext/2022/35010/educating_for_quality_transgender_health_care__a.7.aspx (Accessed November 17, 2024) 10.4103/efh.efh_508_20 36367027

[17] Katz-Wise SL Hyde JS. Sexual fluidity and related attitudes and beliefs among young adults with a same-gender orientation. Arch Sex Behav. (2014) 44:1459–70. doi: 10.1007/s10508-014-0420-1, PMID: 25378265

[18] Filice E Meyer SB. Patterns, predictors, and outcomes of mental health service utilization among lesbians, gay men, and bisexuals: a scoping review. J Gay Lesbian Ment Health. (2018) 22:162–95. doi: 10.1080/19359705.2017.1418468

[19] Lerner JE Robles G. Perceived barriers and facilitators to health care utilization in the United States for transgender people: a review of recent literature. J Health Care Poor Underserved. (2017) 28:127–52. doi: 10.1353/hpu.2017.0014, PMID: 28238993

[20] Bucholc M. The anti-LGBTIQ campaign in Poland: the established, the outsiders, and the legal performance of exclusion. Law Policy. (2022) 44:4–22. doi: 10.1111/lapo.12183

[21] Annual Review 2024. Available online at: https://www.ilgaeurope.org/report/annual-review-2024/ (Accessed August 25, 2024)

[22] Webankieta. Available online at: https://www.webankieta.pl/ (Accessed April 11, 2024)

[23] Crisp C. The gay affirmative practice scale (GAP): a new measure for assessing cultural competence with gay and lesbian clients. Soc Work. (2006) 51:115–26. doi: 10.1093/sw/51.2.115, PMID: 16858917

[24] Karniej P Dissen A Juárez-Vela R Santolalla-Arnedo I Sufrate-Sorzano T Garrote-Camara ME. Psychometric properties and cultural adaptation of the polish version of the gay affirmative practice scale. Front Public Health. (2024) 12:12. doi: 10.3389/fpubh.2024.1384429, PMID: 38756887 PMC11097662

[25] Karniej P Dissen A Juárez-Vela R Martinez Sabater A Pozo-Herce P Gea-Caballero V. Cultural adaptation and psychometric properties of the Spanish version of the gay affirmative practice scale (GAP-ES). Healthcare. (2024) 12:2258. doi: 10.3390/healthcare12222258, PMID: 39595456 PMC11593571

[26] Karniej P Dissen A Juarez-Vela R Gea-Caballero V Echániz-Serrano E Czapla M. Psychometric properties and cultural adaptation of the polish version of the lesbian, gay, bisexual, and transgender development of clinical skills scale (LGBT-DOCSS-PL). J Homosex. (2024) 72:45–59. doi: 10.1080/00918369.2024.2302970, PMID: 38266174

[27] R: The R project for statistical computing. Available online at: https://www.r-project.org/ (Accessed September 27, 2024)

[28] Nowaskie D. A national survey of U.S. psychiatry residents’ LGBT cultural competency: the importance of LGBT patient exposure and formal education. J Gay Lesbian Ment Health. (2020) 24:375–91. doi: 10.1080/19359705.2020.1774848, PMID: 40101104

[29] Council of Europe. New report highlights inadequate healthcare access for LGBTI people and recommends solutions – sexual orientation and gender identity. Available online at: https://www.coe.int/en/web/sogi/-/new-report-highlights-inadequate-healthcare-access-for-lgbti-people-and-recommends-solutions-1 (Accessed November 17, 2024)

[30] Holt E. Supporting Poland’s LGBT+ community through thick and thin. Lancet HIV. doi: 10.1016/S2352-3018(24)00261-3 39341217

[31] Krok A Kardasz Z Rogowska AM. Network analysis of the association between minority stress and activism in LGB people from Poland. Eur J Investig Health Psychol Educ. (2024) 14:1853–67. doi: 10.3390/ejihpe14070122, PMID: 39056637 PMC11276398

[32] Kardasz Z Gerymski R Parker A. Anxiety, attachment styles and life satisfaction in the polish LGBTQ+ community. Int J Environ Res Public Health. (2023) 20:6392. doi: 10.3390/ijerph20146392, PMID: 37510624 PMC10379665

[33] Cole HS Barrow MG Strickland H Robinson S. Empowering nursing students through inclusivity training for LGBTQIA+ patients: a quasi-experimental study. Nurse Educ Today. (2024) 142:106345. doi: 10.1016/j.nedt.2024.106345, PMID: 39128401

[34] May JT Rainbow JG. A qualitative description of direct Care Workers of Lesbian, gay, bisexual, transgender older adults. J Appl Gerontol. (2023) 42:597–606. doi: 10.1177/07334648221139477, PMID: 36384328

[35] Hollinsaid NL Price MA Hatzenbuehler ML. Transgender-specific adolescent mental health provider availability is substantially lower in states with more restrictive policies. J Clin Child Adolesc Psychol. (2024) 53:828–39. doi: 10.1080/15374416.2022.2140433, PMID: 36369805

[36] Walter-McCabe H Chen A. Editors’ introduction: transgender health equity and the law. J Law Med Ethics. (2022) 50:401–8. doi: 10.1017/jme.2022.83, PMID: 36398648 PMC9679591

[37] Council of Europe. New report reveals that LGBTI individuals face inadequate healthcare access and recommends solutions. Centro di Ateneo per i Diritti Umani. (2024). Available online at: https://unipd-centrodirittiumani.it/en/news/council-of-europe-new-report-reveals-that-lgbti-individualsface-inadequate-healthcare-access-and-recommends-solutions (Accessed November 17, 2024)

[38] Balandan K. Mental health inequity and disparity in LGBTQI youth. J Nurse Pract. (2023) 19:104640. doi: 10.1016/j.nurpra.2023.104640

[39] Yu H Flores DD Bonett S Bauermeister JA. LGBTQ + cultural competency training for health professionals: a systematic review. BMC Med Educ. (2023) 23:558. doi: 10.1186/s12909-023-04373-3, PMID: 37559033 PMC10410776

[40] Nair JM Waad A Byam S Maher M. Barriers to care and root cause analysis of LGBTQ+ patients’ experiences: a qualitative study. Nurs Res. (2021) 70:417–24. doi: 10.1097/NNR.0000000000000541, PMID: 34262007

[41] Budge SL Adelson JL Howard KAS. Anxiety and depression in transgender individuals: the roles of transition status, loss, social support, and coping. J Consult Clin Psychol. (2013) 81:545–57. doi: 10.1037/a0031774, PMID: 23398495

[42] Kanakubo Y Sugiyama Y Yoshida E Aoki T Mutai R Matsushima M. Development and validation of the Japanese version of the lesbian, gay, bisexual, and transgender development of clinical skills scale. PLoS One. (2024) 19:e0298574. doi: 10.1371/journal.pone.0298574, PMID: 38536808 PMC10971768

[43] Lantos D Mole RCM Golec de Zavala A. Born this way? National collective narcissism, implicit homophobia, and homosexual essentialism in populist Poland. Arch Sex Behav. (2024) 53:3907–3924. doi: 10.1007/s10508-024-02952-z 39152322 PMC11588881

[44] Fricke J Siddique SM Aysola J Cohen ME Mull NK. Healthcare worker implicit bias training and education: Rapid review. In: Making healthcare safer IV: A continuous updating of patient safety harms and practices. Rockville (MD): Agency for Healthcare Research and Quality (US) (2024). 38330158

[45] Mole RCM de Zavala AG Ardag MM. Homophobia and national collective narcissism in populist Poland. Eur J Sociol Arch Eur Sociol. (2021) 62:37–70. doi: 10.1017/S0003975621000072

[46] Ferrari F Imperato C Mancini T. Heteronormativity and the justification of gender hierarchy: investigating the archival data from 16 European countries. Front Psychol. (2021) 12:686974. doi: 10.3389/fpsyg.2021.686974, PMID: 34393913 PMC8359921

[47] Torres JL Gonçalves GP Pinho AA Souza MHN. The *Brazilian LGBT+ health survey*: Methodology and descriptive results. Available online at: https://www.scielo.br/j/csp/a/wJQNMDdWdz5BjwY3G376b4R/?lang=en (Accessed November 17, 2024) 10.1590/0102-311X00069521 34669766

[48] Wang YC Miao NF You MH. Attitudes toward, knowledge of, and beliefs regarding providing care to LGBT patients among student nurses, nurses, and nursing educators: a cross-sectional survey. Nurse Educ Today. (2022) 116:105472. doi: 10.1016/j.nedt.2022.105472, PMID: 35834866

[49] Cruciani G Quintigliano M Mezzalira S Scandurra C Carone N. Attitudes and knowledge of mental health practitioners towards LGBTQ+ patients: a mixed-method systematic review. Clin Psychol Rev. (2024) 113:102488. doi: 10.1016/j.cpr.2024.102488, PMID: 39168053

[50] Affuso G Picone N Costa PA Bacchini D de Angelis G Esposito C. Minority stress and mental health in gay and lesbian youth: a comparative study of Italy and Spain. Am J Orthopsychiatry. (2024) 94:148–58. doi: 10.1037/ort0000709, PMID: 37883020

[51] Schweiger-Whalen L Noe S Lynch S Summers L Adams E. Converging cultures: partnering in affirmative and inclusive health Care for Members of the lesbian, gay, bisexual, and transgender community. J Am Psychiatr Nurses Assoc. (2019) 25:453–66. doi: 10.1177/1078390318820127, PMID: 30580674

